# Multi-shot diffusion tensor imaging in the lumbosacral spinal cord: Characterizing heterogeneity in healthy tissue and differences in multiple sclerosis

**DOI:** 10.1162/IMAG.a.1296

**Published:** 2026-07-08

**Authors:** Alicia E. Cronin, Xinyu Zhang, Anna Combes, Simon Vandekar, Gabriella L. Dunay, Lipika Narisetti, Grace Sweeney, Logan Prock, Delaney Houston, Aimee Salakhov, Kurt G. Schilling, Seth Stubblefield, Colin D. McKnight, Francesca Bagnato, Subramaniam Sriram, Seth A. Smith, Kristin P. O’Grady

**Affiliations:** Vanderbilt University Institute of Imaging Science, Vanderbilt University Medical Center, Nashville, TN, United States; Department of Radiology and Radiological Sciences, Vanderbilt University Medical Center, Nashville, TN, United States; Department of Biostatistics, Vanderbilt University, Nashville, TN, United States; NMR Research Unit, Queen Square Multiple Sclerosis Centre, UCL Queen Square Institute of Neurology, University College London, London, United Kingdom; Department of Biomedical Engineering, Vanderbilt University, Nashville, TN, United States; Neuroimmunology Division, Department of Neurology, Vanderbilt University Medical Center, Nashville, TN, United States; Department of Neurology, Nashville VA Medical Center, TN Valley Healthcare System, Nashville, TN, United States

**Keywords:** spinal cord, magnetic resonance imaging, multiple sclerosis, diffusion tensor imaging

## Abstract

The clinical presentation of lower extremity and autonomic dysfunction of persons with multiple sclerosis (pwMS) does not consistently correlate with central nervous system pathology noted on conventional magnetic resonance imaging (MRI). Diffusion tensor imaging (DTI) could provide more information about tissue microstructure and insight into pathophysiology but is understudied in relevant anatomies such as the lumbosacral spinal cord (LSC). We sought to explore how DTI measures change across LSC spinal levels and white matter (WM) columns in 29 persons with mild relapsing-remitting MS (pwRRMS) (Expanded Disability Status Scale Median [Min, Max] = 1 [0, 3.5]) and 27 age- and sex-matched healthy controls (HCs) at 3T and evaluate associations between DTI measures and clinical measures of disability. In HCs, differences across spinal levels and between WM columns were found in all DTI measures. However, there were no significant differences when comparing HC and normal-appearing tissue in pwRRMS. In pwRRMS, increased dorsal WM fractional anisotropy (FA) was associated with worse mobility and sensation. We observed heterogeneity in DTI measures throughout the LSC, emphasizing the importance of region-based analysis for future studies. In the LSC, higher FA may not reflect increased microstructural integrity, but expansion to MS populations with greater disability needs to be explored.

## 1 Introduction

Multiple sclerosis (MS) is a chronic inflammatory autoimmune disease that can cause demyelination and neurodegeneration in both the brain and spinal cord (SC) (Hemond & Bakshi, 2018; Kearney, Miller, et al., 2015). Around 80% of persons with MS (pwMS) develop lesions throughout the SC (Bot et al., 2004), which can lead to a variety of neurological deficits affecting quality of life, including motor and gait dysfunction, disturbance in coordination, and bowel and bladder dysfunction (Kearney, Miller, et al., 2015). Advanced imaging of the SC is of interest to further understand the pathological substrates of disability in MS.

In contrast to conventional magnetic resonance imaging (MRI) where MS lesions can be observed, diffusion-weighted imaging (DWI) can provide information about tissue microstructure and anatomical connectivity within the central nervous system. Diffusion tensor imaging (DTI) can provide the quantitative indices fractional anisotropy (FA), axial diffusivity (AD), radial diffusivity (RD), and mean diffusivity (MD), which are sensitive to tissue microstructure features such as fiber integrity and direction, axonal integrity, and degree of myelination (Beaulieu, 2002). DTI could provide a means to detect subtle tissue microstructure changes in pwMS, such as damage to SC white matter (WM), even when conventional MRI does not reveal overt lesions (By et al., 2017; Toosy et al., 2014).

Alterations in tissue microstructure and neurodegeneration have been observed using DTI in the cervical SC of pwMS (Combes et al., 2022; Hesseltine et al., 2006; Naismith et al., 2013; Toosy et al., 2014). While DTI in the lumbosacral SC (LSC) could provide additional insight into MS pathophysiology, to our knowledge, recent DTI studies in the LSC have only been performed in healthy cohorts (Bosma & Stroman, 2012; Büeler et al., 2024; Cronin et al., 2025; Wilm et al., 2009; Yiannakas et al., 2016). This discrepancy largely reflects the technical challenges associated with imaging the LSC. Challenges that arise include magnetic field inhomogeneities due to the surrounding vertebrae and proximity to the lungs, cerebrospinal fluid (CSF) pulsation, and sensitivity to subject motion and physiological motion like respiration (Figley et al., 2008; Stroman et al., 2014).

In pwMS, at least 75% experience bladder dysfunction (Browne et al., 2015), and within 10 years of disease duration, 59% have mild or greater gait disability (Kister et al., 2013). Bladder and gait dysfunction arise from pathology affecting complex neural circuitry that involves the brain, spans multiple spinal cord levels, and includes peripheral pathways. Within this circuitry, the LSC, and more specifically the lumbosacral enlargement (LSE), plays a critical role in gait and bladder dysfunction. Probing tissue microstructure and characterizing DTI measures of the LSE have the potential to provide novel markers of neurological impairment and further our understanding of the sensorimotor and autonomic dysfunction experienced by pwMS, which could ultimately improve clinical management of this disease. Given the functional specialization of the SC, both tract-wise and level-wise, exploring group differences across different regions could help explain specific clinical symptoms.

In this study, a diffusion-weighted acquisition with a reduced field-of-view (FOV) 2D-navigated multi-shot echo planar imaging (EPI) readout (termed image reconstruction using image-space sampling function (IRIS) (Jeong et al., 2013) zonally magnified oblique multi-slice (ZOOM) (Wheeler-Kingshott et al., 2002)) was used in the LSC of healthy controls (HCs) and persons with relapsing-remitting MS (pwRRMS). We sought to determine whether (1) DTI indices are heterogeneous within and throughout the healthy LSC; (2) DTI measures differ between HC and pwRRMS, healthy tissue, normal-appearing tissue, and lesioned tissue; and (3) LSC DTI measures for pwRRMS are related to sensorimotor function.

## 2 Methods

### 2.1 Study participants

The Vanderbilt University Institutional Review Board approved this study, and written informed consent was acquired prior to participation. Thirty-five pwRRMS according to the revised 2017 McDonald Criteria (Thompson et al., 2018) and 29 sex- and age-matched HCs were enrolled. Inclusion criteria for patients included an Expanded Disability Status Scale (EDSS) score ≤4.0 determined by clinical examination, not experiencing an active relapse or receiving an acute corticosteroid treatment 2 weeks prior to recruitment, and known or suspected SC involvement documented during routine clinical assessments or in previous clinical imaging findings. HCs had no previous neurological conditions and were MRI compatible.

### 2.2 Clinical assessments

A sensorimotor examination was completed by participants, which included Timed Up and Go (Nilsagard et al., 2007) (TUG) (average time of two trials), Timed 25-Foot Walk (T25w) (Inojosa et al., 2020) (average time of two trials), and lower extremity vibration threshold measurements using a Vibration-II device (Newsome et al., 2011). Vibration measurements were performed bilaterally on the big toe using a descending method of limits. Bladder symptoms were self-reported based on the Lower Urinary Tract Dysfunction Research Network (LURN) Symptom Index (SI) (LURN SI-10 and LURN SI-29, questions in Supplementary Materials) (Cella et al., 2020). The forms evaluate the impact of urinary symptoms on the quality of life. The score ranges from 0 to 58, with higher scores indicating more severe symptoms.

### 2.3 MR imaging

Imaging was performed on a whole-body 3T Philips dStream Ingenia MR scanner (Philips, Best, Netherlands) with a 2-channel body coil for signal transmission and an integrated 12-channel posterior SC array for reception. For spinal level registration and axial FOV positioning, a sagittal T2-weighed turbo spin echo sequence was acquired (repetition time (TR)/echo time (TE) = 2765/120 ms, resolution = 1.0 x 1.1 mm^2^, slice thickness = 5 mm, number of slices = 11, FOV = 270 x 270 mm^2^, sensitivity encoding (SENSE) = 1.4, and number of signal averages (NSA) = 2). Axial images for all subjects were centered at the LSE and angled to maximize the number of slices perpendicular to the cord. High-resolution axial multi-slice, multi-echo gradient echo (mFFE) images were acquired, and the image resulting from averaging the three echoes was used for spinal level template registration and lesion delineation. The mFFE parameters were TR/TE/ΔTE = 401/6.4/7.0 ms, resolution = 0.65 x 0.65 mm^2^, slice thickness = 5 mm, number of slices = 14, FOV = 150 x 150 mm^2^, flip angle = 43°, NSA = 3, and total scan time of 7 minutes and 27 seconds. The cardiac-triggered, multi-slice, spin-echo diffusion acquisition had the following parameters: IRIS ZOOM multi-shot EPI readout (reduced FOV with a 2D navigation acquired with each shot for motion and phase correction (Jeong et al., 2013; Wheeler-Kingshott et al., 2002)), effective b-value = 750 s/mm^2^ (including a b = 0 s/mm^2^ and 15 diffusion-weighted images in uniformly distributed directions with phase encoding in the right-left direction), TR = 5 beats (~5000 ms), TE = 48 ms, echo train length = 22 (2 shots), resolution = 1.1 x 1.1 mm^2^, slice thickness = 5 mm, number of slices = 14, FOV = 80 x 58 mm^2^, flip angle = 90°, SENSE = 1, partial Fourier half scan factor = 0.6, NSA = 1, total scan time of ~8–11 minutes (depending on heart rate).

### 2.4 Image processing

Processing was performed using FMRIB Software Library (FSL) v6.0.7.11 (Jenkinson et al., 2012), Spinal Cord Toolbox (SCT) v6.2 (De Leener et al., 2017), MRTrix3 v3.0.4 (Tournier et al., 2019), and in-house code. Preprocessing of the diffusion data began with denoising using the Marcenko-Pastur PCA algorithm (Veraart et al., 2016) in MRTrix3, and slice-wise registration-based motion correction using SCT (*sct_dmri_moco*) with regularization along the rostral-caudal axis (polynomial order of 2) (Xu et al., 2013). The *eddy* and *topop* FSL algorithms were not included in the preprocessing due to a previous study reporting that these distortion correction techniques did not consistently correct the geometry within the SC (Schilling et al., 2024). In SCT, the diffusion tensor was fit voxel-wise to the denoised, motion-corrected diffusion data using standard least squares fitting (*sct_dmri_compute_dti*), which produced FA, AD, RD, and MD maps. To register the b = 0 image and the anatomical mFFE, slice-wise center of mass alignment of the cord segmentation and rigid registration using Mutual Information between the images (*sct_register_multimodal*) were used.

On the b = 0 image, whole SC segmentation of the image volume was performed using SCT (*sct_deepseg*) and manually corrected as needed. After averaging three diffusion images with good contrast between GM and WM, the GM was manually outlined on the average image, as demonstrated previously (Kearney, Schneider, et al., 2015; Yiannakas et al., 2016). To quantify the reproducibility of this approach, A.E.C. and G.L.D. independently outlined the GM in HCs and the Dice Similarity Coefficient (DSC) was calculated per slice and averaged over all participants. WM masks were created by subtracting the GM from SC masks, and dorsal, ventral, and lateral (right and left) column masks were created. In the pwRRMS cohort, lesion masks were manually drawn on the mFFE image (average of three echoes) by Dr. McKnight (11 years of neuroradiology experience) and Dr. Stubblefield (4 years of diagnostic radiology experience) independently, and a DSC was calculated per slice and averaged over all pwRRMS. A consensus lesion mask was created, with more weight given to the more experienced rater after two MR imaging scientists (A.E.C. and K.P.O.) identified and removed lesion mask voxels attributed to imaging artifacts associated with mFFE images. The final consensus mask was used for the subsequent analyses. To assess lesion distribution in WM and GM, lesion masks were multiplied by GM and WM masks to obtain GM and WM lesions, respectively. Using the transformation matrices from the diffusion and mFFE registration, the lesion masks were transformed into diffusion space. Using SCT, template spinal levels were registered to the sagittal T2-weighted image, where spinal level refers to the neurological SC level, and co-registered to the mFFE and diffusion. Slice-wise diffusion measurements were averaged within each spinal level and tissue type.

### 2.5 Statistical analysis

Linear mixed-effects models were used to evaluate the effects of biological variables (age, sex, weight, height), spinal level (T12–S3), and tissue region (GM, dorsal WM, ventral WM, and lateral WM) on the DTI indices in each tissue region within the HCs, including a random effect for participant. Spinal levels with greater than nine observations were included. The effect of biological variables was evaluated across spinal levels by including interactions between spinal level and each biological variable. To compare pwRRMS normal-appearing tissue and HCs, separate mixed-effects linear models were fit on the region-based DTI measures with age, sex, group (HC or pwRRMS), and group-by-spinal-level interactions included as covariates, testing the interaction effect.

To compare lesions with normal-appearing pwRRMS and HC tissue, lesions and DTI measures were collapsed into two spinal level categories (due to the low number of lesions in each individual spinal level): upper levels (T12–L4) and lower levels (L5–S3). Linear mixed-effects models were fit on the dorsal, lateral, and GM DTI measures with lesion status and spinal level category as covariates, and the interactions tested for comparisons between lesions and HC tissue (between-subject) and normal-appearing pwRRMS tissue (within-subject). The ventral region was omitted due to few lesions. For all models, Type 2 sum-of-squares tests were used, so that main effects were tested without their interaction terms in the model.

For pwRRMS, we investigated relationships between DTI measures in normal-appearing tissue and measures of mobility, great toe vibration sensitivity (right-left average), bladder symptoms, and disease duration. We conducted four models with DTI indices as the outcome measure and primary covariates of mobility (Timed Up and Go and Timed 25-Foot Walk), vibration sensation, bladder score, or disease duration, with sex, age, and collapsed spinal levels (upper and lower) as additional covariates for each. For the bladder score model, we excluded three pwRRMS with documented non-MS-related urological comorbidities (two pelvic floor dysfunction and one prostate enlargement) to minimize potential confounding bladder symptoms. This resulted in n = 19 pwRRMS with bladder scores for the bladder–DTI association analysis. For the mobility measures, we computed an aggregate mobility time index by Z-scoring both mobility tests across all participants and averaging the Z-scores for each participant. Multicollinearity among clinical covariates was assessed using pair-wise correlations and no evidence of excessive multicollinearity was observed. Specifically, age showed a moderate correlation with disease duration (r = 0.589) and mobility score (r = 0.448). For all statistical analyses, AD, MD, and RD values were multiplied by 1000 for interpretability, while FA values were kept on the original scale. For all statistical tests, FDR (Benjamini–Hochberg) correction was applied. All statistics were performed in R version 4.3.3 (R Foundation for Statistical Computing, Vienna, Austria).

## 3 Results

### 3.1 Study participants

Six pwRRMS and two HCs were excluded from further analysis due to severe motion artifact on the diffusion or anatomical image. The final sample was 29 pwRRMS and 27 HCs; complete demographic details are given in Table 1 for the final sample. Note that the cohorts remain age- and sex-matched following the exclusion. Comparative statistical descriptors between groups for the clinical measures given in Table 1 are provided for context only.

**Table 1.** Demographics and clinical measures for healthy controls and persons with relapsing-remitting multiple sclerosis.

	HC (n = 27)	pwRRMS (n = 29)	p-value
**Sex**	20F/7M	23F/6M	NS
**Age (years)**			
Mean ± SD	39.4 ± 13.4	41.9 ± 12.1	NS
Median [Min, Max]	35.9 [21.9, 66.4]	39.6 [21.0, 64.1]	
**Height (inches)**			
Mean ± SD	65.3 ± 4.48	66.8 ± 3.72	NS
Median [Min, Max]	65.0 [59.0, 76.0]	67.0 [59.0, 75.0]	
**Weight (pounds)**			
Mean ± SD	152 ± 34	172 ± 39.4	0.04*
Median [Min, Max]	140 [110, 226]	165 [97, 251]	
**Disease Duration (years)**			
Mean ± SD	n/a	12.1 ± 9.41	n/a
Median [Min, Max]	n/a	8.60 [0.17, 33.9]	
**EDSS**			
Mean ± SD	n/a	1.32 ± 1.47	n/a
Median [Min, Max]	n/a	1 [0, 3.5]	
Missing	n/a	18	
**T25w (s)**			
Mean ± SD	4.55 ± 0.52	5.57 ± 1.27	0.0006***
Median [Min, Max]	4.75 [3.46, 5.27]	5.34 [3.53, 9.18]	
Missing	12	0	
**TUG (s)**			
Mean ± SD	6.55 ± 0.75	8.35 ± 1.90	<0.0001****
Median [Min, Max]	6.39 [5.46, 7.60]	8.05 [5.01, 13.9]	
Missing	12	0	
**Vibration Threshold**			
Mean ± SD	1.41 ± 0.57	2.3 ± 1.26	0.004**
Median [Min, Max]	1.24 [0.70, 2.54]	2.08 [0.75, 5.32]	
Missing	12	4^a^	
**Bladder Score**			
Mean ± SD	5.20 ± 6.27	10.4 ± 7.73	0.03*
Median [Min, Max]	3 [0, 26]	8 [1, 27]	
Missing	12	7	

All comparisons were performed with Student’s t-tests, except sex was compared with the chi-square test.

a Four patients did not have vibration data due to an inability to feel the vibration at the starting point.

*p < 0.05, **p < 0.01, ***p < 0.001, ****p < 0.0001.

F = female, M = male, EDSS = Expanded Disability Status Scale, T25w = Timed 25-Foot Walk, TUG = Timed Up and Go, SD = Standard Deviation, n/a = not applicable, HC = healthy control, pwRRMS = persons with relapsing remitting multiple sclerosis, NS = non-significant.

### 3.2 White matter regional heterogeneity in HCs

**Fig. 1.**
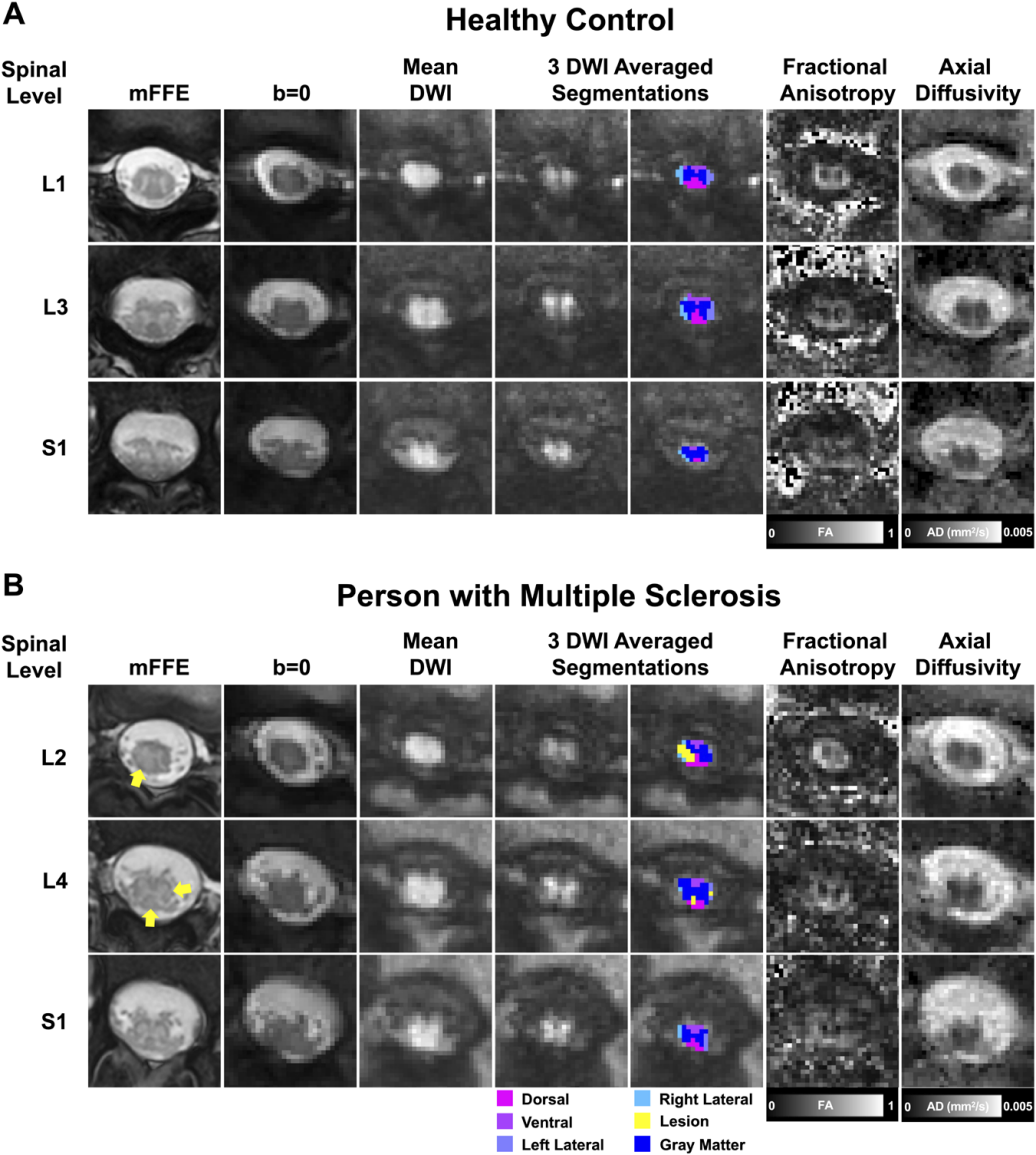
Example data for one healthy control (A, 26-year-old female) and one person with multiple sclerosis (B, 42-year-old female). Shown are the anatomical multi-echo, gradient echo (mFFE) images, image with no diffusion-sensitizing gradients (b = 0), mean diffusion-weighted image (DWI), three DWI slices averaged with good gray matter (GM) contrast without (left) and with (right) GM, white matter columns, and lesions overlaid, and the fractional anisotropy (FA) and axial diffusivity (AD) quantitative maps. All images are shown for three slices corresponding to spinal levels throughout the lumbar enlargement. Lesions are highlighted by the yellow area on the anatomical slices.

**Fig. 2.**
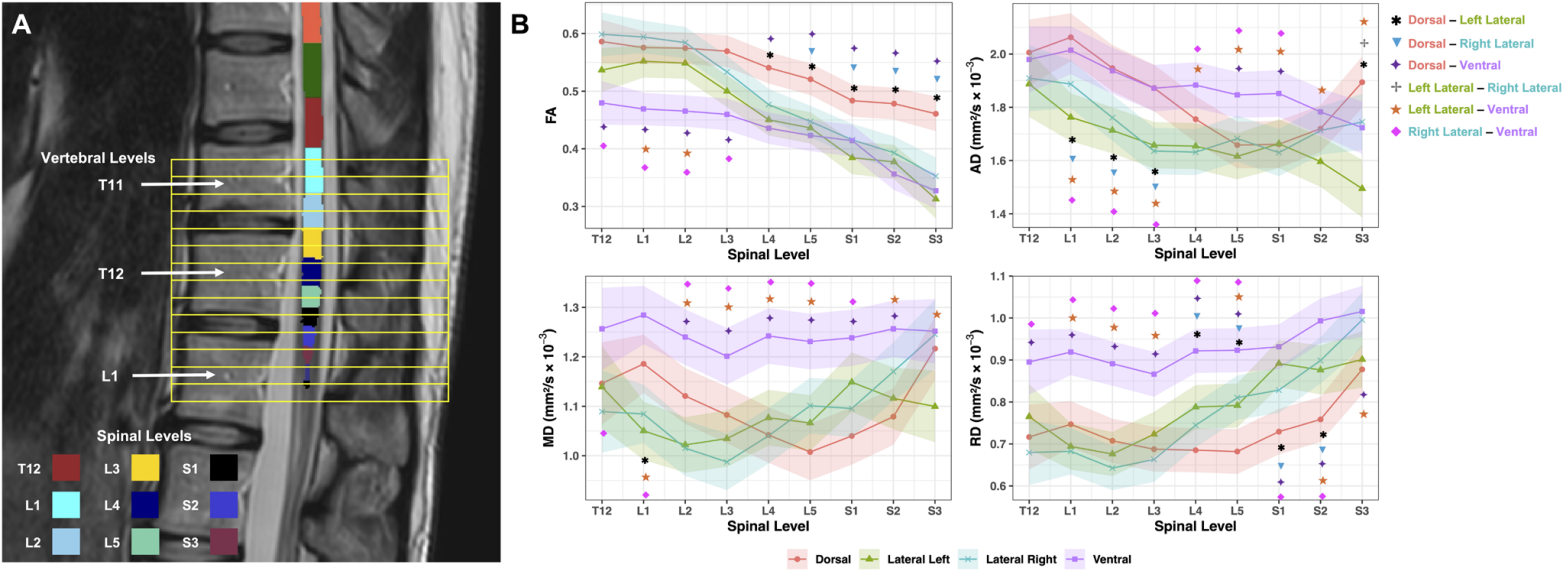
(A) Representative T2-weighted sagittal image of one healthy participant (33-year-old female), with axial diffusion field-of-view (FOV) overlaid in yellow with registered spinal levels. The lumbosacral (neurological) spinal levels typically correspond spatially with vertebral levels ~T11–L1, though there is some inter-subject variability that is accounted for when registering template spinal levels to individual subject images. (B) Marginal mean by white matter column (dorsal, left lateral, right lateral, and ventral) in DTI measures fractional anisotropy (FA), axial diffusivity (AD), radial diffusivity (RD), and mean diffusivity (MD) across all spinal levels in healthy controls. Shapes (✱▼✦✢★◆) indicate an FDR-adjusted p-value (correction with Benjamini–Hochberg method across spinal levels) < 0.05 for the marginal mean difference between the different white matter columns for a given spinal level.

Inter-rater agreement for the HCs GM segmentation was moderate to good (DSC = 0.74 ± 0.08). Representative anatomical and diffusion images for three spinal level slices along the LSE for both an HC and pwRRMS are shown in Figure 1, with an example of the axial FOV and corresponding spinal levels shown in Figure 2A. For all DTI measures, there were significant spinal level effects. The marginal means of each WM column (dorsal, left lateral, right lateral, and ventral) in the HCs for each DTI measure across the spinal levels are shown in Figure 2B. In all DTI measures, there were no significant differences between the left and right lateral columns (excluding level S3 for AD; Supplementary Table S1). However, there were significant differences between the ventral, dorsal, and lateral columns for almost all the spinal levels in all DTI measures. Because there were no significant differences between the left and right lateral columns, these were collapsed into one “lateral” category for subsequent analysis.

### 3.3 Biological variability in HCs

In HCs, we aimed to determine whether biological variables had any significant associations with DTI measures, considering the interactions at different spinal levels (Supplementary Tables S2–S5). There were no main or interaction effects for height or sex. For age, there was a significant interaction effect in the dorsal column for AD ($\chi^{2}$ = 21.37, p = 0.006, p_FDR_ = 0.025), but no significant main effect. For weight, there was a significant interaction effect in the dorsal column for FA ($\chi^{2}$ = 21.14, p = 0.007, p_FDR_ = 0.027), but no main effect. The significant interaction effect was due to a directional change across spinal levels (Supplementary Fig. S1). However, because MS presentation is influenced by age and sex (Capasso et al., 2023; Golden & Voskuhl, 2017), both were included as covariates moving forward.

### 3.4 Lesion distribution

Inter-rater lesion mask agreement between the two radiologists was DSC = 0.51 ± 0.2, while there was higher, but not perfect, agreement between the more experienced rater’s individual mask and the final consensus mask (DSC = 0.90 ± 0.1), which removed “lesion” voxels attributed to mFFE image artifacts. In the pwRRMS cohort, 28 of 29 patients had a lesion detected in at least one slice between T12 and S3. In the upper spinal level group (T12–L4), 65.73% of the slices had a lesion in one type of tissue. Specifically, 50.4% of slices had a dorsal lesion, 39.92% had a lateral lesion, and 33.47% had a GM lesion. In the lower spinal level group (L5–S3), 19.2% of slices had a lesion in any type of tissue, with a dorsal lesion in 10.0% of slices, a lateral lesion in 9.23% of slices, and a GM lesion in 15.38% of slices. Throughout the whole lumbar SC, 36.51%, 29.37%, and 27.25% of slices had a dorsal, lateral, and GM lesion, respectively. Table 2 summarizes the distribution of lesions throughout the lumbar SC.

**Table 2.** Number of slices that have lesions and the total number of slices for the different tissue types (dorsal, lateral, and gray matter) for each spinal level group (T12–L4 and L5–S3) in the relapsing-remitting cohort (n = 29).

	Dorsal (# of slices with lesion/total slices)	Lateral (# of slices with lesion/total slices)	Gray matter (# of slices with lesion/total slices)	Total (# of slices with lesion/total slices)
**T12–L4**	125/248 (50.40%)	99/248 (39.92%)	83/248 (33.47%)	163/248 (65.73%)
**L5–S3**	13/130 (10.0%)	12/130 (9.23%)	20/130 (15.38%)	25/130 (19.23%)
**Total**	138/378 (36.51%)	111/378 (29.37%)	103/378 (27.25%)	188/378 (49.73%)

### 3.5 Differences between normal-appearing pwRRMS and HC tissue

**Fig. 3.**
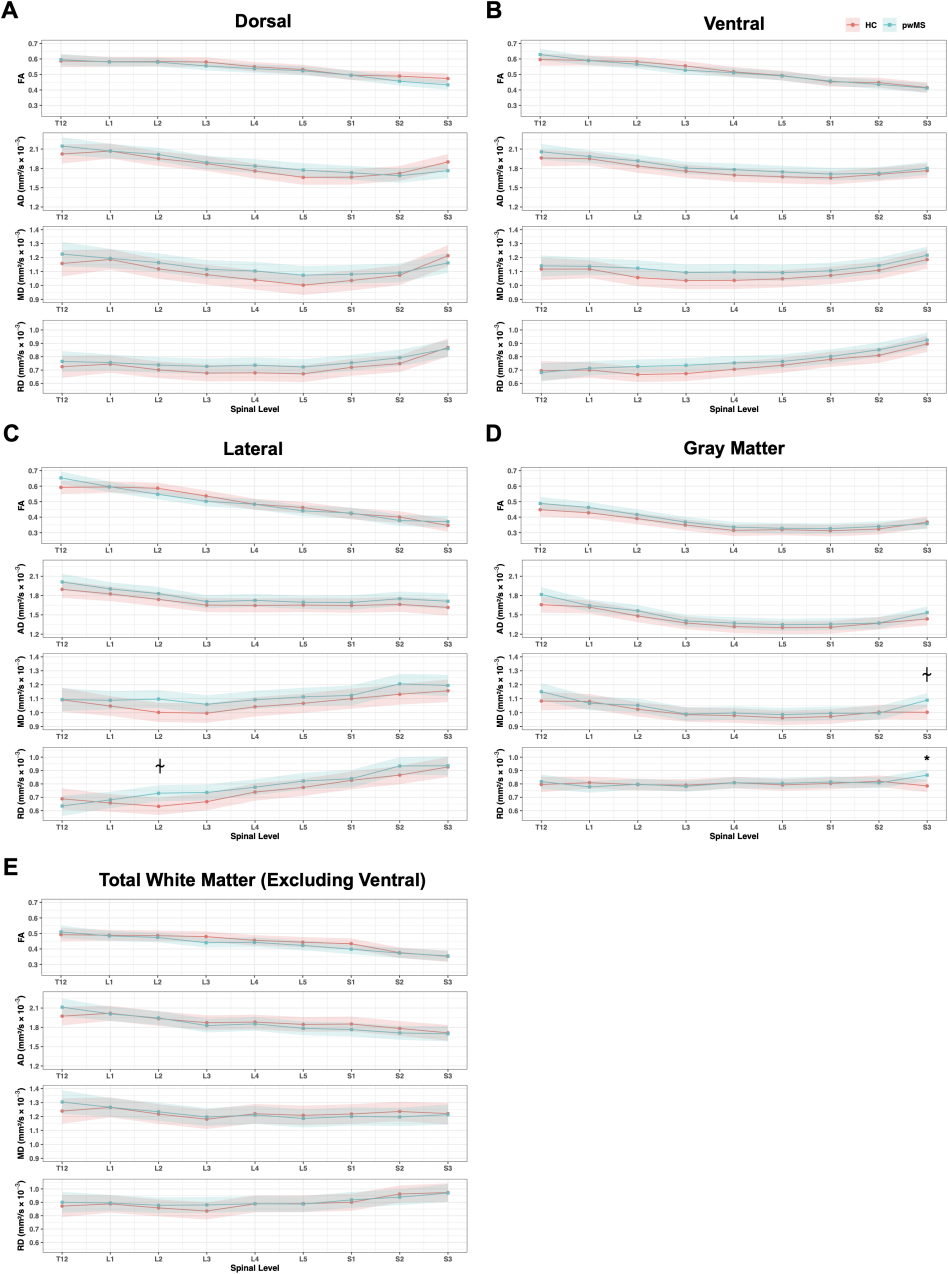
Marginal means for healthy controls (HCs) and persons with relapsing-remitting multiple sclerosis (pwRRMS) in the normal-appearing white matter columns dorsal (A), ventral (B), lateral (C), gray matter (D), and total white matter excluding ventral (E) in DTI measures fractional anisotropy (FA), axial diffusivity (AD), radial diffusivity (RD), and mean diffusivity (MD) across all spinal levels. * Indicates an FDR-adjusted p-value (correction with Benjamini–Hochberg method across spinal levels) < 0.05. ⍭ Indicates an FDR-adjusted p-value (correction with Benjamini–Hochberg method across spinal levels) < 0.10.

We tested group differences (between HCs and normal-appearing pwRRMS tissue) in each spinal level using FDR-adjusted p-values within each DTI measurement (Fig. 3; Table 3). The only significant marginal mean difference between groups was at the S3 level in GM tissue for the RD measurement (p = 0.004; p_FDR_ = 0.034). The only significant interaction effect was in the lateral column for the FA measurement ($\chi^{2}$ = 24.98, p = 0.002; p_FDR_ = 0.006), and no significant main group effects.

**Table 3.** Unadjusted and FDR-adjusted p-values (correction with Benjamini–Hochberg method across spinal levels within each DTI measure) for the marginal mean group differences between healthy controls and persons with multiple sclerosis (normal-appearing tissue) for all spinal levels.

		Spinal levels								
DTI		T12	L1	L2	L3	L4	L5	S1	S2	S3
**Dorsal**										
FA	p	0.732	0.920	0.831	0.161	0.502	0.652	0.957	0.071	0.041
	p_FDR_	0.957	0.957	0.957	0.482	0.957	0.957	0.957	0.320	0.320
AD	p	0.191	0.998	0.343	0.806	0.254	0.097	0.329	0.597	0.067
	p_FDR_	0.514	0.998	0.514	0.906	0.514	0.437	0.514	0.768	0.437
MD	p	0.265	0.864	0.280	0.359	0.128	0.093	0.301	0.690	0.273
	p_FDR_	0.451	0.864	0.451	0.461	0.451	0.451	0.451	0.777	0.451
RD	p	0.456	0.768	0.327	0.178	0.118	0.174	0.376	0.247	0.838
	p_FDR_	0.586	0.838	0.565	0.535	0.535	0.535	0.565	0.556	0.838
**Ventral**										
FA	p	0.506	0.838	0.568	0.044	0.505	0.297	0.072	0.888	0.904
	p_FDR_	0.852	0.904	0.852	0.324	0.852	0.852	0.324	0.904	0.904
AD	p	0.128	0.891	0.884	0.508	0.669	0.359	0.197	0.299	0.842
	p_FDR_	0.808	0.891	0.891	0.891	0.891	0.808	0.808	0.808	0.891
MD	p	0.261	0.981	0.691	0.711	0.832	0.589	0.679	0.347	0.875
	p_FDR_	0.981	0.981	0.981	0.981	0.981	0.981	0.981	0.981	0.981
RD	p	0.597	0.865	0.598	0.232	0.978	0.933	0.641	0.536	0.935
	p_FDR_	0.978	0.978	0.978	0.978	0.978	0.978	0.978	0.978	0.978
**Lateral**										
FA	p	0.022	0.937	0.053	0.087	0.984	0.297	0.852	0.308	0.325
	p_FDR_	0.195	0.984	0.237	0.261	0.984	0.487	0.984	0.487	0.487
AD	p	0.174	0.226	0.147	0.384	0.201	0.484	0.485	0.203	0.214
	p_FDR_	0.339	0.339	0.339	0.485	0.339	0.485	0.485	0.339	0.339
MD	p	0.971	0.318	0.017	0.110	0.200	0.253	0.588	0.094	0.445
	p_FDR_	0.971	0.477	0.151	0.331	0.451	0.456	0.661	0.331	0.572
RD	p	0.282	0.542	0.008	0.062	0.317	0.201	0.769	0.096	0.846
	p_FDR_	0.475	0.697	0.071	0.280	0.475	0.453	0.846	0.288	0.846
**GM**										
FA	p	0.156	0.113	0.207	0.367	0.315	0.672	0.519	0.434	0.662
	p_FDR_	0.621	0.621	0.621	0.651	0.651	0.672	0.668	0.651	0.672
AD	p	0.042	0.697	0.154	0.588	0.334	0.420	0.393	0.966	0.100
	p_FDR_	0.375	0.784	0.463	0.756	0.630	0.630	0.630	0.966	0.449
MD	p	0.113	0.672	0.347	0.892	0.556	0.448	0.453	0.801	0.008
	p_FDR_	0.507	0.864	0.815	0.892	0.834	0.815	0.815	0.892	0.070
RD	p	0.553	0.245	0.922	0.726	0.981	0.643	0.688	0.622	0.004
	p_FDR_	0.934	0.934	0.981	0.934	0.981	0.934	0.934	0.934	**0.034**
**WM (No Ventral)**										
FA	p	0.180	0.995	0.371	0.126	0.740	0.874	0.794	0.512	0.846
	p_FDR_	0.809	0.995	0.984	0.809	0.984	0.984	0.984	0.984	0.984
AD	p	0.235	0.597	0.178	0.418	0.156	0.212	0.334	0.790	0.565
	p_FDR_	0.528	0.671	0.528	0.626	0.528	0.528	0.602	0.790	0.671
MD	p	0.653	0.606	0.067	0.117	0.102	0.230	0.362	0.365	0.432
	p_FDR_	0.653	0.653	0.352	0.352	0.352	0.518	0.548	0.548	0.556
RD	p	0.770	0.702	0.069	0.061	0.151	0.387	0.516	0.206	0.421
	p_FDR_	0.770	0.770	0.309	0.309	0.454	0.631	0.664	0.464	0.631

Bold values indicate statistically significant results after FDR correction.

DTI = diffusion tensor imaging, FA = fractional anisotropy, AD = axial diffusivity, MD = mean diffusivity, RD = radial diffusivity, WM = white matter.

### 3.6 Differences between lesions and normal-appearing and HC tissue

**Fig. 4.**
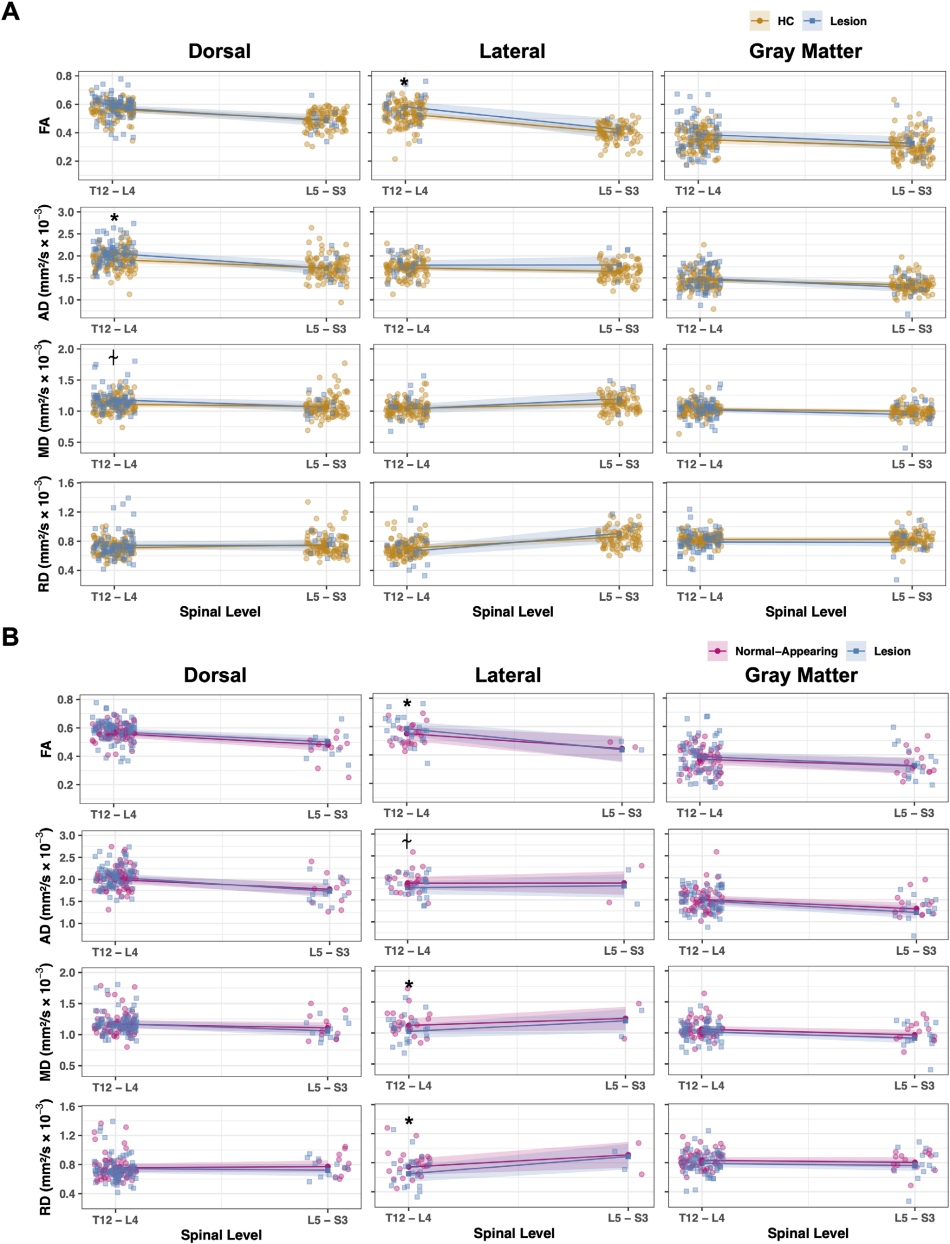
Marginal means for healthy controls (HCs) and corresponding lesioned tissue in persons with relapsing-remitting multiple sclerosis (pwRRMS) (A) and normal-appearing tissue and paired lesioned tissue in pwRRMS (B) for the dorsal and lateral white matter columns and gray matter tissue in DTI measures fractional anisotropy (FA), axial diffusivity (AD), radial diffusivity (RD), and mean diffusivity (MD) across grouped spinal levels. *Indicates an FDR-adjusted p-value (correction with Benjamini–Hochberg method) < 0.05. ⍭ Indicates an FDR-adjusted p-value (correction with Benjamini–Hochberg method) < 0.10.

Main and interaction effect results for lesion tissue in pwRRMS versus either the corresponding HC tissue or the paired normal-appearing pwRRMS tissue across the grouped spinal levels (T12–L4, L5–S3) for all DTI measures are shown in Table 4. There were no main effects or interactions between HC tissue and lesions, but there were significant main effects between the paired normal-appearing lateral WM and lateral WM lesions in pwRRMS for all DTI measurements. For comparisons between HC tissue and pwRRMS lesions for the upper and lower spinal levels (Fig. 4A), there was a significant marginal mean difference in the upper spinal levels for dorsal WM AD (p_FDR_ = 0.015) and for lateral WM FA (p_FDR_ = 0.049). For comparisons between paired normal-appearing tissue and lesions in pwRRMS (Fig. 4B), the marginal mean difference was significant for lateral WM FA (p_FDR_ = 0.035), MD (p_FDR_ = 0.010), and RD (p_FDR_ = 0.007) within the upper spinal levels (T12–L4).

**Table 4.** Main lesion status effect, and interaction effect between lesion status and spinal levels on all DTI measures for dorsal and lateral white matter and gray matter.

		Main lesion status effect					Interaction effect				
	DTI	$\chi^{2}$	DF	Effect size	p	p_FDR_	$\chi^{2}$	DF	Effect size	p	p_FDR_
**HC vs pwRRMS lesioned tissue**	**Dorsal**										
	FA	0.196	1	0.000	0.658	0.658	0.314	1	0.000	0.575	0.575
	AD	5.157	1	0.221	0.023	0.093	3.176	1	0.160	0.075	0.299
	MD	3.255	1	0.163	0.071	0.142	1.911	1	0.104	0.167	0.334
	RD	1.054	1	0.025	0.305	0.406	0.634	1	0.000	0.426	0.568
	**Lateral**										
	FA	4.845	1	0.236	0.028	0.111	0.561	1	0.000	0.454	0.474
	AD	1.556	1	0.090	0.212	0.425	0.512	1	0.000	0.474	0.474
	MD	0.000	1	0.000	0.994	0.994	1.655	1	0.097	0.198	0.397
	RD	0.780	1	0.000	0.377	0.503	2.109	1	0.127	0.146	0.397
	**GM**										
	FA	3.696	1	0.176	0.055	0.156	0.059	1	0.000	0.808	0.832
	AD	0.107	1	0.000	0.743	0.743	0.640	1	0.000	0.424	0.832
	MD	1.062	1	0.027	0.303	0.404	0.772	1	0.000	0.380	0.832
	RD	3.110	1	0.156	0.078	0.156	0.045	1	0.000	0.832	0.832
**Paired pwRRMS normal-appearing vs lesioned tissue**	**Dorsal**										
	FA	2.266	1	0.199	0.132	0.306	0.163	1	0.000	0.686	0.686
	AD	1.854	1	0.163	0.173	0.306	1.209	1	0.081	0.272	0.511
	MD	0.004	1	0.000	0.952	0.952	1.436	1	0.117	0.231	0.511
	RD	1.446	1	0.118	0.229	0.306	0.759	1	0.000	0.383	0.511
	**Lateral**										
	FA	5.542	1	0.550	0.019	**0.023**	0.770	1	0.000	0.380	0.790
	AD	5.169	1	0.527	0.023	**0.023**	0.057	1	0.000	0.811	0.811
	MD	9.088	1	0.734	0.003	**0.005**	0.286	1	0.000	0.593	0.790
	RD	9.468	1	0.751	0.002	**0.005**	0.460	1	0.000	0.497	0.790
	**GM**										
	FA	1.198	1	0.077	0.274	0.274	0.176	1	0.000	0.674	0.964
	AD	1.343	1	0.103	0.247	0.274	0.346	1	0.000	0.556	0.964
	MD	4.523	1	0.327	0.033	0.067	0.084	1	0.000	0.771	0.964
	RD	4.816	1	0.340	0.028	0.067	0.002	1	0.000	0.964	0.964

Bold values indicate statistically significant results after FDR correction.

DTI = diffusion tensor imaging, FA = fractional anisotropy, AD = axial diffusivity, MD = mean diffusivity, RD = radial diffusivity, GM = gray matter, pwRRMS = persons with relapsing-remitting multiple sclerosis, DF = degree of freedom.

### 3.7 Associations with sensorimotor measures

**Fig. 5.**
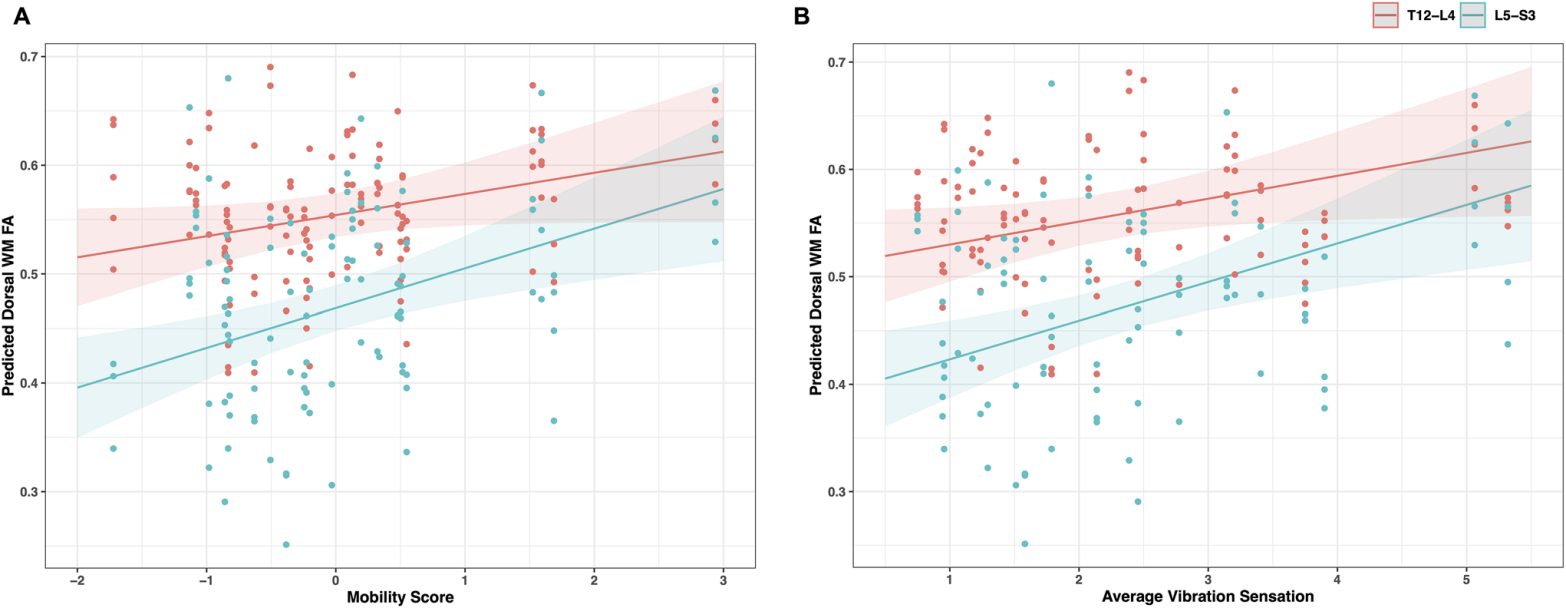
(A) Model-predicted dorsal white matter (WM) fractional anisotropy (FA) across the full range of mobility scores for both spinal level groups (T12–L4, L5–S3). For T12–L4, the β = 0.0194, with 95% confidence intervals (CI) of [-0.00151, 0.0404]. For L5–S3, the β = 0.0365, with 95% CI of [0.0151, 0.0579]. (B) Model-predicted dorsal WM FA across vibration sensation for both spinal level groups. For T12–L4, the β = 0.0214, with 95% confidence intervals (CI) of [-0.0004, 0.0428]. For L5–S3, the β = 0.0359, with 95% CI of [0.01413, 0.0577].

Main and interaction effects for normal-appearing pwRRMS tissue versus the sensorimotor measures across the grouped spinal levels for all DTI measures are shown in Supplementary Tables S6–S8. We did not find evidence for interaction effects across upper/lower spinal levels. There were significant main effects between dorsal WM FA and both vibration sensation ($\chi^{2}$ = 8.83, p = 0.003, p_FDR_ = 0.012) and mobility score ($\chi^{2}$ = 8.95, p = 0.003, p_FDR_ = 0.011) when controlling for sex, age, and spinal level. Figure 5 shows the predicted trends of each, demonstrating that higher dorsal FA is associated with worse sensation and mobility. In the lateral WM, there was a significant main effect between FA and mobility before the correction ($\chi^{2}$ = 5.71, p = 0.017, p_FDR_ = 0.067). Finally, disease duration and bladder score did not have any significant main effects.

## 4 Discussion

Building on our previous work developing multi-shot LSC DTI with high anatomical fidelity and signal-to-noise ratio (Cronin et al., 2025), we sought to assess spinal level and tissue region heterogeneity, differences between HC, normal-appearing, and lesioned tissue, and relationships between DTI and clinical measures. Differences between WM columns and LSC spinal levels were observed within HCs, indicating that evaluating DTI measures by spinal level and columns is valuable, but there were no significant main effects of group (disease status) in models comparing HC tissue and normal-appearing tissue in pwRRMS. Within pwRRMS, DTI measures (FA, MD, and RD) differed significantly between lateral WM lesions and paired normal-appearing lateral WM tissue within upper spinal levels (levels T12–L4 combined). Finally, higher FA in normal-appearing dorsal WM was associated with higher vibration threshold scores (worse sensation) and worse mobility scores. It should be noted that this study explores the DTI metrics from an acquisition with 15 diffusion-weighted directions, chosen to balance tensor estimation accuracy with scan time feasibility when using the cardiac-triggered, 2D-navigated reduced FOV multi-shot EPI approach.

Although evaluating the LSC with MRI is of interest to understand pathological substrates of lower extremity and bladder dysfunction in pwMS, to our knowledge, prior DTI studies of this anatomy have focused only on healthy cohorts (Bosma & Stroman, 2012; Büeler et al., 2024; Cronin et al., 2025; Wilm et al., 2009; Yiannakas et al., 2016). To understand how DTI metrics could relate to clinical symptoms, we first needed to determine how DTI measures vary throughout the healthy LSC and between WM columns. Büeler et al. (2024) explored the scan–rescan reliability of DTI measurements throughout the LSE and within the various WM columns, but did not investigate differences between WM column measurements. Instead, they used the slice with the largest GM area as the LSE landmark and examined the reproducibility of three equal segments on each side. In our study, we explored the heterogeneity in DTI indices across WM columns and spinal levels.

Heterogeneity in DTI indices was found along and within the WM of the healthy LSC, possibly due to the different types of ascending and descending tracts in each. While the left and right lateral WM columns are functionally distinct due to the crossing of the nerve tracts, our DTI measures did not detect microstructural differences between the columns, which may be due to a lack of sensitivity in DTI. Partial volume effects could also contribute to differences between WM columns. With the current image resolution and the large proportion of GM in the LSC, contamination from GM is possible even with manually edited GM ROIs. Furthermore, the ventral WM column is small compared with the other regions and contains the median fissure, which may cause partial volume effects from CSF. All DTI measures also changed across spinal levels, which could be due to the changing proportion of WM to GM (i.e., the enlargement has more WM than the sacral region) (Henmar et al., 2020) and differences in tract density and orientation. Partial volume effects would also be more prominent in the sacral levels due to both the small cross-section and acquisition slice thickness, contributing to differences in DTI measures across the spinal levels. However, previous studies have also shown that DTI indices significantly differ throughout the spinal levels of the cervical SC (Chan et al., 2015; Ellingson et al., 2008) and the slices/segments of the LSC (Büeler et al., 2024; Cronin et al., 2025).

While many MS studies have focused on the cervical SC, the LSC is comparatively understudied. This is despite reports that ~40% of SC focal lesions and 60% of diffuse SC changes occur in the broader thoracolumbar region (Weier et al., 2012), which encompasses the lower thoracic SC and distal spinal levels. In our study, 96.5% of patients had an LSC lesion identified in anatomical imaging. Although larger than previously reported numbers in the lumbar region (Bussas et al., 2022), the T2*-weighted mFFE used in this study is more sensitive to lesions than conventional T2-weighted imaging (Martin et al., 2012), which was used in the previous study (Bussas et al., 2022). More lesions were also identified in the upper levels of the LSC, which may be due to either the larger SC area increasing the ability to distinguish lesions or increased lesion frequency. Our findings support the notion that SC lesions are predominantly found in the dorsal and lateral columns (Kreiter et al., 2024); however, the lower spinal levels had a greater proportion of GM lesions, which may be due to the smaller area of WM than GM in these levels. When comparing the findings of lesion prevalence in the LSC from this study with other studies, it is important to keep in mind the differences in patient inclusion (i.e., patients had known or suspected SC involvement) and sensitivity to lesions in mFFE acquisitions compared with conventional T2-weighted images.

The presence (or absence) of lesions does not fully explain clinical presentation using conventional MRI (Stankiewicz et al., 2009); however, advanced MRI techniques used to examine normal-appearing tissue in the cervical SC have shown more substantial and significant correlations with motor function (Naismith et al., 2013; Zackowski et al., 2009), suggesting that microstructural damage may extend beyond focal lesions and across SC levels. Since the SC is somatotopically organized, WM columns contain tracts related to specific body functions, and examining column-specific associations with DTI measures could further assist our understanding of clinical disability. Vibration sensation is specific to the dorsal column, which carries proprioceptive information, and in pwRRMS, great toe sensation is crucial for maintaining static standing balance (Chou et al., 2009). Because the dorsal columns are crucial for proprioception, damage could lead to a loss of coordination and balance, affecting mobility. Findings in our study demonstrate that higher FA in normal-appearing WM of the dorsal columns is associated with worse sensation and mobility. Naismith et al. (2013) found that in the cervical SC, DTI measures were significantly correlated with vibration sensation in the dorsal columns, while mobility was slower with decreased FA in both the dorsal and lateral columns. While our results do not align with these prior observations in the cervical SC (Naismith et al., 2013), this may suggest that in the LSC, higher FA may not necessarily reflect preserved microstructural integrity. This counterintuitive relationship between higher dorsal FA and worse mobility and sensation could reflect the strong influence of tissue geometry in the LSC and the strong dependence of FA on geometry. Decreased directional dispersion within fibers can elevate FA even when underlying pathology is present, and increased FA does not necessarily reflect increased fiber density or preserved microstructural integrity. Similar observations have been reported in the corticospinal tract, where FA was unchanged or increased in MS due to tract curvature dominating the FA signal in the periventricular region (Reich et al., 2007).

When we controlled for spinal level interactions and included age and sex as covariates, we found no differences between DTI indices in HC tissue and the corresponding normal-appearing pwRRMS tissue. When comparing the HCs with the lesioned tissue, the dorsal AD and lateral FA measures of lesions were significantly greater than those in the WM of the HCs. In the lateral columns of pwRRMS, the lesioned tissue had higher FA than normal-appearing tissue, while normal-appearing tissue had higher MD and RD than the lesioned tissue. These unexpected findings merit careful interpretation. Elevated FA in the lateral lesions, with a concurrent reduction in MD and RD, could suggest reorganized tissue rather than simple demyelination. Preferential loss of crossing fibers in non-dominant orientations would increase coherence (i.e., higher FA) while preserving longitudinal tract organizations that restrict perpendicular diffusion (i.e., lower RD). This pattern aligns with histopathological validation using neurite orientation dispersion and density imaging (NODDI) in the post-mortem MS SC, which demonstrated reduced orientation dispersion within demyelinated lesions by both diffusion and histology (Grussu et al., 2017). This suggests that there may be a reduction or loss in collateral branching or non-dominant crossing fibers in MS lesions, which could cause an increase in FA.

Because these trends differ from previous reports in the cervical SC (Combes et al., 2022; Hesseltine et al., 2006; Naismith et al., 2013; Toosy et al., 2014), lesion effects in the LSC require further exploration. Regional differences between the cervical SC and LSC may also explain divergence. A previous study in the LSC has demonstrated that the lower SC had lower FA and AD, and higher RD, than the cervical spinal cord (Yiannakas et al., 2016), suggesting a more complex and dispersed fiber orientation. This may cause the LSC to be more susceptible to degeneration of crossing pathways while preserving more of the longitudinal tract organization. The lesion differences observed in the present study could be due to partial volume effects, as some lesions are only a single voxel in diffusion space, and effects of normal-appearing and lesion tissue may be mixed. Furthermore, compared with the normal-appearing tissue, the number of lesion samples is significantly lower and increasing the sample size should be explored. Future studies should use orientation-specific metrics (i.e. fiber orientation distribution functions) and NODDI to directly assess whether elevated FA in lesions reflects genuine axonal loss in the non-dominant orientations. Advanced diffusion models and multi-shell acquisitions could help interpret changes in myelination, fiber density, and crossing fibers independently, establishing whether LSC lesions have distinct pathophysiology compared with the brain and cervical SC.

Limitations of this study to consider include the relatively low disability level of our pwRRMS population (inclusion criteria of EDSS ≤4.0). Future work with primary progressive and secondary progressive MS participants, who typically exhibit more clinical symptoms, could further our understanding of the associations between DTI indices in the LSC and sensorimotor measures. This study also focused on participants with known or suspected SC involvement, likely increasing the observed SC lesion prevalence compared with a general MS population. Therefore, the high proportion of patients in this cohort with at least 1 lesion (28/29, or 96.5%) should not be generalized to all pwRRMS. Moderate agreement between the two independent raters also needs to be considered, suggesting variability in lesion identification. While the consensus approach incorporated both neuroradiologists’ and MR imaging scientists’ reviews, the reported lesion prevalence should be interpreted with caution. A future study should incorporate automated segmentation of lesions in the LSC and compare with manually drawn lesions, but this is beyond the scope of the current study as automated methods for this anatomy and T2*-weighted image contrast are still under development. Furthermore, a limitation of our lesion analysis is the binary grouping of spinal levels to achieve adequate statistical power when comparing lesions with normal-appearing tissue and sensorimotor associations. We were unable to characterize DTI differences in lesions at the individual spinal levels due to statistical power, which should be performed in future studies with larger sample sizes.

Additionally, missing bladder symptom scores (n = 7) could have affected our association analysis. Patients with missing bladder assessments may represent a distinct subgroup with different clinical characteristics, which may introduce bias into our association analysis. Our cohort also exhibited heterogeneity in disease duration (range 0.17–33.9 years). While stratifying by disease duration could help characterize how DTI metrics evolve along the disease trajectory and whether disease stages affect SC pathology and microstructure as MS typically progresses with disease chronicity, this was not feasible due to the sample size constraints of our current cohort. Future studies with larger sample sizes should consider disease stage-specific analyses.

Finally, as mentioned, partial volume effects between tissue types (WM and GM lesions and normal-appearing tissue) and spinal levels must be considered in the LSC, particularly in the sacral levels due to the resolution of the current diffusion acquisition. It has been shown that spinal level S3 is important for bladder innervation (Carlucci et al., 2014), and partial volume effects from adjacent levels within the image slice that includes S3 may prevent significant associations with bladder function from being shown. It should also be noted that while three pwRRMS were identified and excluded with documented non-MS-related urological comorbidities, prospective screening for such comorbidities at study entry would strengthen future investigations. Future studies should aim to increase axial resolution (decrease slice thickness) while maintaining a reasonable scan time, thereby improving lesion localization accuracy in diffusion images and enabling further investigation into lesion differences in the LSC. This would allow further evaluation of whether DTI metrics in both lesions and normal-appearing tissue explain bladder function.

## 5 Conclusion

The findings from this study indicate that in the healthy LSC, DTI indices in WM columns differ from one another and across spinal levels, cautioning against the averaging of DTI measures across different levels and columns for group comparisons. No significant differences were found between normal-appearing pwRRMS tissue and HC tissue indices throughout the LSC; however, some differences were observed between normal-appearing tissue and lesions. Additional work is needed to explore whether these differences have pathological relevance. Future applications of DTI to MS populations with greater disability will help assess whether LSC DTI can be used as an objective, quantifiable measure of SC pathology and disease progression.

## Supplementary Material

Supplementary Material

## Data Availability

Raw data and processing scripts are available from the corresponding author upon reasonable request.
