# Supplementary material for: Multi-shot diffusion tensor imaging in the lumbosacral spinal cord: Characterizing heterogeneity in healthy tissue and differences in multiple sclerosis
Source: Imaging Neurosci (Camb). 2026 Jul 8;4:IMAG.a.1296. doi: 10.1162/IMAG.a.1296 (PMC13347604; doi:10.1162/IMAG.a.1296)
Supplement: Supplementary Material [file IMAG.a.1296_supp.pdf]

Supplementary Materials

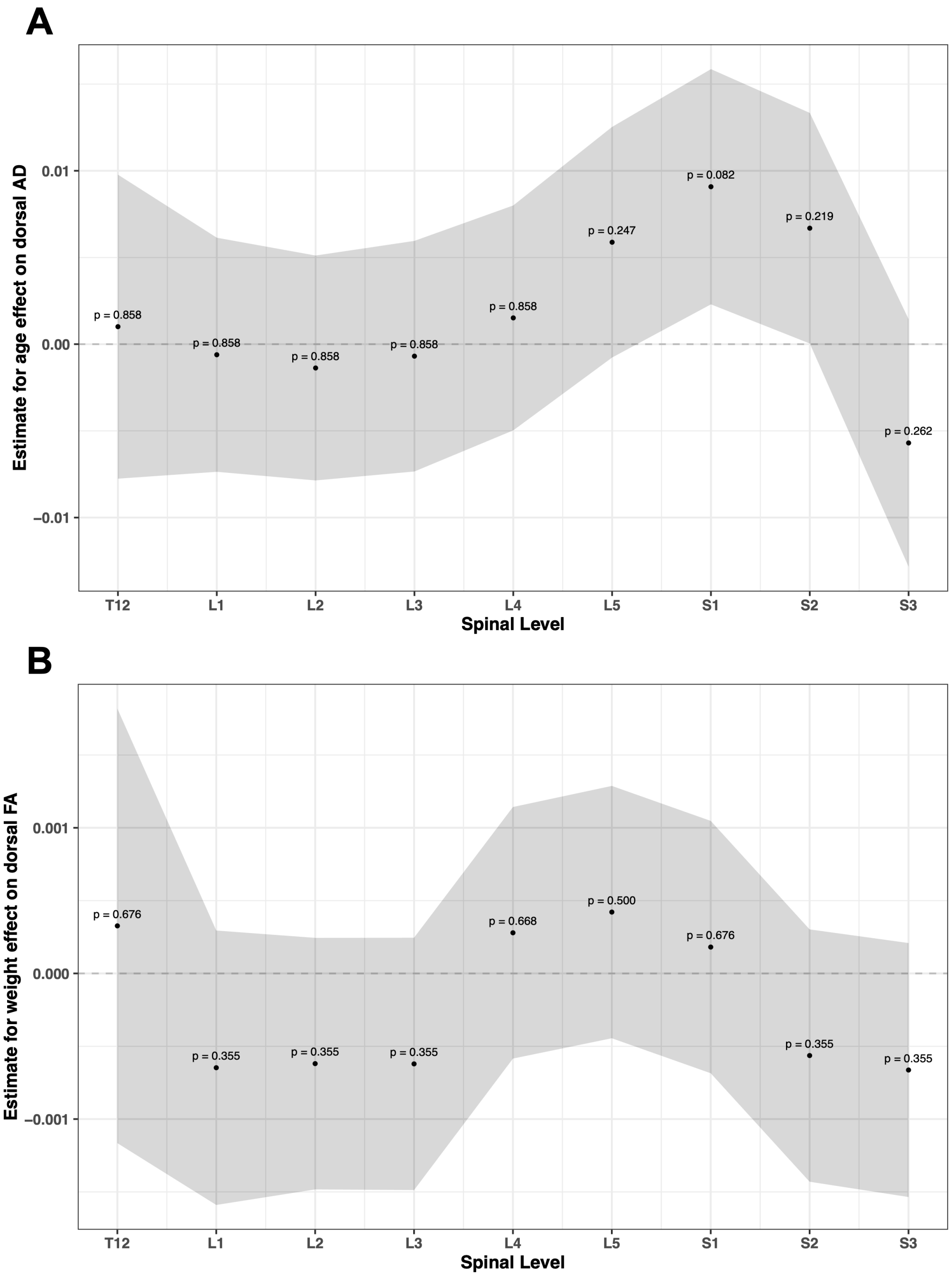

Supplementary Figure 1: Estimated effect of **A)** age on axial diffusivity (AD) and **B)** body weight on fractional anisotropy (FA) across all spinal levels (T12-S3) in healthy controls. Each point represents estimated slopes at each spinal level and shaded areas represent 95% confidence intervals. No significant associations were observed (non-significant FDR-corrected adjusted p-values).

Supplementary Table 1: Unadjusted and FDR-adjusted p-values (correction with Benjamini-Hochberg method) for the marginal mean group differences between white matter columns within healthy controls for all spinal levels and DTI measures.

		Spinal Level								
		**T12**	**L1**	**L2**	**L3**	**L4**	**L5**	**S1**	**S2**	**S3**
**FA**										
dorsal - latL	p	0.172	0.471	0.336	0.000	0.000	0.000	0.000	0.000	0.000
	p_{FDR}	0.221	0.471	0.378	0.000	0.000	0.000	0.000	0.000	0.000
dorsal - latR	p	0.955	0.676	0.925	0.105	0.000	0.000	0.000	0.000	0.000
	p_{FDR}	0.955	0.869	0.955	0.157	0.000	0.000	0.000	0.000	0.000
dorsal - ventral	p	0.000	0.000	0.000	0.000	0.000	0.000	0.000	0.000	0.000
	p_{FDR}	0.000	0.000	0.000	0.000	0.000	0.000	0.000	0.000	0.000
latL - latR	p	0.051	0.051	0.099	0.148	0.322	0.890	0.236	0.789	0.213
	p_{FDR}	0.231	0.231	0.296	0.332	0.414	0.890	0.354	0.887	0.354
latL - ventral	p	0.089	0.000	0.000	0.053	0.783	0.833	0.265	0.584	0.883
	p_{FDR}	0.200	0.000	0.000	0.159	0.883	0.883	0.477	0.877	0.883
latR - ventral	p	0.000	0.000	0.000	0.000	0.041	0.401	1.000	0.100	0.563
	p_{FDR}	0.000	0.000	0.000	0.000	0.074	0.515	1.000	0.150	0.633
**AD**										
dorsal - latL	p	0.473	0.000	0.000	0.000	0.217	0.852	1.000	0.126	0.000
	p_{FDR}	0.608	0.000	0.000	0.001	0.325	0.959	1.000	0.227	0.000
dorsal - latR	p	0.643	0.009	0.002	0.000	0.085	0.968	0.940	0.999	0.097
	p_{FDR}	0.965	0.026	0.010	0.001	0.175	0.999	0.999	0.999	0.175
	p	0.989	0.818	0.998	1.000	0.069	0.002	0.002	0.637	0.029

dorsal - ventral	p_{FDR}	1.000	1.000	1.000	1.000	0.155	0.011	0.011	1.000	0.087
latL - latR	p	0.993	0.111	0.799	0.978	0.972	0.577	0.931	0.178	0.002
	p_{FDR}	0.993	0.499	0.993	0.993	0.993	0.993	0.993	0.535	0.016
latL - ventral	p	0.674	0.000	0.000	0.000	0.000	0.000	0.004	0.005	0.003
	p_{FDR}	0.674	0.000	0.000	0.001	0.000	0.000	0.005	0.006	0.005
latR - ventral	p	0.827	0.101	0.004	0.000	0.000	0.010	0.000	0.570	0.987
	p_{FDR}	0.930	0.151	0.010	0.000	0.000	0.017	0.001	0.733	0.987
**MD**										
dorsal - latL	p	0.999	0.002	0.031	0.561	0.767	0.369	0.023	0.774	0.048
	p_{FDR}	0.999	0.021	0.093	0.841	0.871	0.664	0.093	0.871	0.109
dorsal - latR	p	0.743	0.040	0.018	0.048	1.000	0.050	0.447	0.072	0.916
	p_{FDR}	0.955	0.112	0.112	0.112	1.000	0.112	0.671	0.129	1.000
dorsal - ventral	p	0.205	0.049	0.005	0.007	0.000	0.000	0.000	0.000	0.840
	p_{FDR}	0.230	0.062	0.010	0.010	0.000	0.000	0.000	0.000	0.840
latL - latR	p	0.814	0.809	0.998	0.571	0.741	0.767	0.503	0.521	0.012
	p_{FDR}	0.916	0.916	0.998	0.916	0.916	0.916	0.916	0.916	0.108
latL - ventral	p	0.158	0.000	0.000	0.000	0.000	0.000	0.085	0.002	0.005
	p_{FDR}	0.158	0.000	0.000	0.000	0.000	0.000	0.095	0.003	0.007
latR - ventral	p	0.016	0.000	0.000	0.000	0.000	0.002	0.001	0.097	0.999
	p_{FDR}	0.021	0.000	0.000	0.000	0.000	0.003	0.001	0.109	0.999
**RD**										
dorsal - latL	p	0.792	0.443	0.782	0.721	0.012	0.007	0.000	0.006	0.942
	p_{FDR}	0.891	0.797	0.891	0.891	0.026	0.020	0.000	0.020	0.942
dorsal - latR	p	0.893	0.266	0.210	0.890	0.284	0.001	0.023	0.000	0.023

	p_{FDR}	0.893	0.365	0.365	0.893	0.365	0.004	0.052	0.003	0.052
dorsal - ventral	p	0.004	0.000	0.000	0.000	0.000	0.000	0.000	0.000	0.003
	p_{FDR}	0.004	0.000	0.000	0.000	0.000	0.000	0.000	0.000	0.003
latL - latR	p	0.360	0.988	0.747	0.291	0.565	0.944	0.281	0.926	0.144
	p_{FDR}	0.811	0.988	0.988	0.811	0.988	0.988	0.811	0.988	0.811
latL - ventral	p	0.062	0.000	0.000	0.000	0.000	0.001	0.668	0.007	0.037
	p_{FDR}	0.070	0.000	0.000	0.001	0.001	0.001	0.668	0.010	0.047
latR - ventral	p	0.000	0.000	0.000	0.000	0.000	0.004	0.015	0.034	0.963
	p_{FDR}	0.000	0.000	0.000	0.000	0.000	0.006	0.019	0.039	0.963

latL = lateral left; latR = lateral right, DTI = diffusion tensor imaging, FA = fractional anisotropy, AD = axial diffusivity, MD = mean diffusivity, RD = radial diffusivity

Supplementary Table 2: Main sex effect and interaction effect between sex and spinal levels on all DTI measures for white matter columns and gray matter in healthy controls.

	Main Sex Effect					Interaction Effect				
**DTI**	χ^2	**DF**	**Effect Size**	**p**	p_{FDR}	χ^2	**DF**	**Effect Size**	**p**	p_{FDR}
**Dorsal**										
FA	0.068	1	0	0.795	0.795	10.69	8	0.315	0.220	0.881
AD	0.618	1	0	0.432	0.795	2.25	8	0.000	0.972	0.972
MD	0.492	1	0	0.483	0.795	3.01	8	0.000	0.934	0.972
RD	0.171	1	0	0.679	0.795	6.34	8	0.000	0.609	0.972
**Ventral**										
FA	0.010	1	0	0.920	0.955	8.92	8	0.185	0.349	0.349
AD	0.003	1	0	0.955	0.955	9.14	8	0.206	0.330	0.349
MD	0.067	1	0	0.796	0.955	9.93	8	0.267	0.270	0.349
RD	0.133	1	0	0.715	0.955	10.78	8	0.321	0.215	0.349
**Lateral**										
FA	0.308	1	0	0.579	0.895	9.31	8	0.220	0.317	0.713
AD	0.293	1	0	0.589	0.895	6.12	8	0.000	0.633	0.713
MD	0.056	1	0	0.813	0.895	5.41	8	0.000	0.713	0.713
RD	0.017	1	0	0.895	0.895	7.06	8	0.000	0.530	0.713
**GM**										
FA	0.043	1	0	0.836	0.918	6.84	8	0.000	0.554	0.554
AD	0.342	1	0	0.559	0.918	7.77	8	0.000	0.456	0.554
MD	0.095	1	0	0.758	0.918	8.17	8	0.079	0.417	0.554
RD	0.011	1	0	0.918	0.918	7.67	8	0.000	0.466	0.554

	Main Sex Effect					Interaction Effect				
**DTI**	χ^2	**DF**	**Effect Size**	**p**	p_{FDR}	χ^2	**DF**	**Effect Size**	**p**	p_{FDR}
**WM (No Ventral)**										
FA	0.208	1	0	0.648	0.900	12.32	8	0.400	0.137	0.550
AD	0.454	1	0	0.501	0.900	1.56	8	0.000	0.992	0.992
MD	0.175	1	0	0.675	0.900	2.98	8	0.000	0.936	0.992
RD	0.003	1	0	0.953	0.953	8.13	8	0.070	0.421	0.841

DTI = diffusion tensor imaging, FA = fractional anisotropy, AD = axial diffusivity, MD = mean diffusivity, RD = radial diffusivity, GM = gray matter, WM = white matter, DF = degree of freedom

Supplementary Table 3: Main age effect and interaction effect between age and spinal levels on all DTI measures for white matter columns and gray matter in healthy controls.

	**Main Age Effect**					**Interaction Effect**				
**DTI**	χ^2	**DF**	**Effect Size**	**p**	**p_{FDR}**	χ^2	**DF**	**Effect Size**	**p**	**p_{FDR}**
**Dorsal**										
FA	1.672	1	0.158	0.196	0.616	4.19	8	0.000	0.839	0.839
AD	0.759	1	0.000	0.384	0.616	21.37	8	0.704	0.006	0.025
MD	0.029	1	0.000	0.864	0.864	16.19	8	0.551	0.040	0.079
RD	0.542	1	0.000	0.462	0.616	9.07	8	0.200	0.336	0.448
**Ventral**										
FA	4.494	1	0.360	0.034	0.075	8.01	8	0.020	0.432	0.577
AD	0.184	1	0.000	0.668	0.668	5.27	8	0.000	0.728	0.728
MD	1.117	1	0.066	0.291	0.387	8.56	8	0.144	0.381	0.577
RD	4.317	1	0.350	0.038	0.075	10.92	8	0.329	0.206	0.577
**Lateral**										
FA	2.630	1	0.246	0.105	0.210	10.48	8	0.303	0.233	0.567
AD	0.051	1	0.000	0.821	0.821	6.72	8	0.000	0.567	0.567
MD	1.392	1	0.121	0.238	0.317	7.01	8	0.000	0.535	0.567
RD	3.784	1	0.321	0.052	0.207	8.47	8	0.132	0.389	0.567
**GM**										
FA	0.647	1	0.000	0.421	0.685	16.69	8	0.567	0.033	0.134
AD	0.037	1	0.000	0.847	0.847	13.88	8	0.467	0.085	0.170
MD	0.426	1	0.000	0.514	0.685	7.89	8	0.000	0.444	0.461
RD	1.321	1	0.109	0.250	0.685	7.72	8	0.000	0.461	0.461

	Main Age Effect					Interaction Effect				
**DTI**	χ^2	**DF**	**Effect Size**	**p**	**p_{FDR}**	χ^2	**DF**	**Effect Size**	**p**	**p_{FDR}**
**WM (No Ventral)**										
FA	2.456	1	0.232	0.117	0.234	6.07	8	0.000	0.639	0.639
AD	0.612	1	0.000	0.434	0.579	11.29	8	0.349	0.186	0.529
MD	0.248	1	0.000	0.618	0.618	10.01	8	0.273	0.264	0.529
RD	2.578	1	0.242	0.108	0.234	8.23	8	0.092	0.412	0.549

DTI = diffusion tensor imaging, FA = fractional anisotropy, AD = axial diffusivity, MD = mean diffusivity, RD = radial diffusivity, GM = gray matter, WM = white matter, DF = degree of freedom

Supplementary Table 4: Main height effect and interaction effect between height and spinal levels on all DTI measures for white matter columns and gray matter in healthy controls.

	Main Height Effect					Interaction Effect				
**DTI**	χ^2	**DF**	**Effect Size**	**p**	p_{FDR}	χ^2	**DF**	**Effect Size**	**p**	p_{FDR}
**Dorsal**										
FA	0.242	1	0.000	0.623	0.623	15.53	8	0.528	0.050	0.199
AD	0.257	1	0.000	0.612	0.623	4.22	8	0.000	0.836	0.905
MD	0.985	1	0.000	0.321	0.623	3.42	8	0.000	0.905	0.905
RD	1.201	1	0.086	0.273	0.623	7.21	8	0.000	0.514	0.905
**Ventral**										
FA	0.028	1	0.000	0.867	0.867	10.88	8	0.327	0.208	0.208
AD	0.969	1	0.000	0.325	0.483	11.04	8	0.336	0.199	0.208
MD	1.259	1	0.098	0.262	0.483	13.60	8	0.455	0.093	0.186
RD	0.830	1	0.000	0.362	0.483	15.99	8	0.544	0.042	0.170
**Lateral**										
FA	0.046	1	0.000	0.830	0.830	10.98	8	0.332	0.203	0.502
AD	0.363	1	0.000	0.547	0.745	7.67	8	0.000	0.467	0.502
MD	0.552	1	0.000	0.458	0.745	7.33	8	0.000	0.502	0.502
RD	0.342	1	0.000	0.559	0.745	8.55	8	0.142	0.382	0.502
**GM**										
FA	0.579	1	0.000	0.447	0.596	6.92	8	0.000	0.546	0.735
AD	0.028	1	0.000	0.868	0.868	3.60	8	0.000	0.892	0.892
MD	1.364	1	0.116	0.243	0.486	6.86	8	0.000	0.551	0.735
RD	2.539	1	0.239	0.111	0.444	12.21	8	0.395	0.142	0.569

	Main Height Effect					Interaction Effect				
**DTI**	χ^2	**DF**	**Effect Size**	**p**	p_{FDR}	χ^2	**DF**	**Effect Size**	**p**	p_{FDR}
**WM (No Ventral)**										
FA	0.136	1	0.000	0.713	0.713	19.11	8	0.642	0.014	0.057
AD	0.397	1	0.000	0.528	0.705	1.81	8	0.000	0.986	0.986
MD	1.324	1	0.110	0.250	0.535	3.07	8	0.000	0.930	0.986
RD	1.230	1	0.092	0.267	0.535	10.31	8	0.292	0.244	0.488

DTI = diffusion tensor imaging, FA = fractional anisotropy, AD = axial diffusivity, MD = mean diffusivity, RD = radial diffusivity, GM = gray matter, WM = white matter, DF = degree of freedom

Supplementary Table 5: Main weight effect and interaction effect between weight and spinal levels on all DTI measures for white matter columns and gray matter in healthy controls.

	**Main Weight Effect**					**Interaction Effect**				
**DTI**	χ^2	**DF**	**Effect Size**	**p**	p_{FDR}	χ^2	**DF**	**Effect Size**	**p**	p_{FDR}
**Dorsal**										
FA	0.542	1	0.000	0.462	0.616	21.14	8	0.698	0.007	0.027
AD	0.106	1	0.000	0.744	0.744	2.19	8	0.000	0.975	0.975
MD	0.838	1	0.000	0.360	0.616	3.59	8	0.000	0.892	0.975
RD	1.589	1	0.148	0.207	0.616	8.40	8	0.121	0.396	0.791
**Ventral**										
FA	1.269	1	0.100	0.260	0.870	6.87	8	0.000	0.551	0.706
AD	0.375	1	0.000	0.540	0.870	5.47	8	0.000	0.706	0.706
MD	0.004	1	0.000	0.947	0.947	8.70	8	0.161	0.368	0.706
RD	0.203	1	0.000	0.652	0.870	10.95	8	0.330	0.205	0.706
**Lateral**										
FA	0.828	1	0.000	0.363	0.726	8.59	8	0.148	0.378	0.378
AD	0.002	1	0.000	0.962	0.962	19.22	8	0.645	0.014	0.055
MD	0.351	1	0.000	0.553	0.738	14.15	8	0.477	0.078	0.156
RD	0.929	1	0.000	0.335	0.726	10.12	8	0.280	0.257	0.342
**GM**										
FA	0.008	1	0.000	0.927	0.927	7.66	8	0.000	0.467	0.658
AD	0.187	1	0.000	0.666	0.887	4.17	8	0.000	0.841	0.841
MD	0.544	1	0.000	0.461	0.887	7.40	8	0.000	0.494	0.658
RD	0.479	1	0.000	0.489	0.887	11.11	8	0.339	0.196	0.658

	Main Weight Effect					Interaction Effect				
**DTI**	χ^2	**DF**	**Effect Size**	**p**	p_{FDR}	χ^2	**DF**	**Effect Size**	**p**	p_{FDR}
**WM (No Ventral)**										
FA	0.078	1	0.000	0.780	0.951	17.12	8	0.581	0.029	0.115
AD	0.033	1	0.000	0.856	0.951	5.69	8	0.000	0.682	0.682
MD	0.008	1	0.000	0.929	0.951	6.29	8	0.000	0.614	0.682
RD	0.004	1	0.000	0.951	0.951	10.24	8	0.288	0.248	0.497

DTI = diffusion tensor imaging, FA = fractional anisotropy, AD = axial diffusivity, MD = mean diffusivity, RD = radial diffusivity, GM = gray matter, WM = white matter, DF = degree of freedom

Supplementary Table 6: Main and interaction effects between dorsal white matter and spinal levels on all DTI measures for sensorimotor measures in persons with relapsing-remitting multiple sclerosis.

	Main Dorsal Effect					Interaction Effect				
DTI	χ^2	DF	Effect Size	p	p_{FDR}	χ^2	DF	Effect Size	p	p_{FDR}
**Disease**										
FA	1.366	1	0.079	0.243	0.441	0.029	1	0.000	0.865	0.865
AD	0.222	1	0.000	0.637	0.637	0.059	1	0.000	0.809	0.865
MD	0.945	1	0.000	0.331	0.441	0.052	1	0.000	0.819	0.865
RD	1.515	1	0.094	0.218	0.441	0.038	1	0.000	0.845	0.865
**Bladder**										
FA	0.563	1	0.000	0.453	0.539	1.347	1	0.096	0.246	0.408
AD	2.421	1	0.193	0.120	0.473	0.685	1	0.000	0.408	0.408
MD	1.401	1	0.103	0.237	0.473	0.957	1	0.000	0.328	0.408
RD	0.377	1	0.000	0.539	0.539	1.209	1	0.028	0.310	0.408
**Mobility**										
FA	8.947	1	0.370	0.003	0.011	2.955	1	0.184	0.086	0.342
AD	0.007	1	0.000	0.934	0.934	0.161	1	0.000	0.688	0.789
MD	1.406	1	0.084	0.236	0.314	0.072	1	0.000	0.789	0.789
RD	4.900	1	0.259	0.027	0.054	0.599	1	0.000	0.439	0.789
**Vibration sensation**										
FA	8.827	1	0.396	0.003	0.012	2.494	1	0.173	0.114	0.457
AD	0.999	1	0.000	0.318	0.424	0.963	1	0.000	0.326	0.653
MD	0.023	1	0.000	0.880	0.880	0.043	1	0.000	0.835	0.835
RD	1.291	1	0.076	0.256	0.424	0.147	1	0.000	0.701	0.835

DTI = diffusion tensor imaging, FA = fractional anisotropy, AD = axial diffusivity, MD = mean diffusivity, RD = radial diffusivity, DF = degree of freedom

Supplementary Table 7: Main and interaction effects between lateral white matter and spinal levels on all DTI measures for sensorimotor measures in persons with relapsing-remitting multiple sclerosis.

	Main Lateral Effect					Interaction Effect				
DTI	χ^2	DF	Effect Size	p	p_{FDR}	χ^2	DF	Effect Size	p	p_{FDR}
**Disease**										
FA	0.976	1	0.000	0.323	0.431	0.410	1	0.000	0.522	0.736
AD	0.400	1	0.000	0.527	0.527	0.132	1	0.000	0.716	0.736
MD	1.485	1	0.092	0.223	0.431	0.139	1	0.000	0.709	0.736
RD	2.286	1	0.150	0.131	0.431	0.113	1	0.000	0.736	0.736
**Bladder**										
FA	2.409	1	0.193	0.121	0.241	0.412	1	0.000	0.521	0.521
AD	3.051	1	0.232	0.081	0.241	2.092	1	0.169	0.148	0.278
MD	1.192	1	0.071	0.275	0.367	1.824	1	0.147	0.177	0.278
RD	0.069	1	0.000	0.793	0.793	1.581	1	0.124	0.209	0.278
**Mobility**										
FA	5.714	1	0.288	0.017	0.067	0.166	1	0.000	0.684	0.870
AD	0.000	1	0.000	0.991	0.991	0.134	1	0.000	0.714	0.870
MD	1.026	1	0.021	0.311	0.415	0.080	1	0.000	0.777	0.870
RD	3.228	1	0.198	0.072	0.145	0.027	1	0.000	0.870	0.870
**Vibration sensation**										
FA	1.705	1	0.120	0.192	0.677	0.278	1	0.000	0.598	0.791
AD	0.046	1	0.000	0.831	0.831	0.142	1	0.000	0.706	0.791
MD	0.200	1	0.000	0.655	0.831	0.132	1	0.000	0.716	0.791
RD	0.916	1	0.000	0.338	0.677	0.070	1	0.000	0.791	0.791

DTI = diffusion tensor imaging, FA = fractional anisotropy, AD = axial diffusivity, MD = mean diffusivity, RD = radial diffusivity, DF = degree of freedom

Supplementary Table 8: Main and interaction effects between gray matter and spinal levels on all DTI measures for sensorimotor measures in persons with relapsing-remitting multiple sclerosis.

	Main Gray Matter Effect					Interaction Effect				
**DTI**	χ^2	**DF**	**Effect Size**	**p**	p_{FDR}	χ^2	**DF**	**Effect Size**	**p**	p_{FDR}
**Disease**										
FA	0.780	1	0.000	0.377	0.503	0.288	1	0.000	0.592	0.592
AD	0.096	1	0.000	0.757	0.757	0.350	1	0.000	0.554	0.592
MD	1.029	1	0.022	0.310	0.503	0.836	1	0.000	0.360	0.592
RD	2.786	1	0.175	0.095	0.380	1.096	1	0.041	0.295	0.592
**Bladder**										
FA	0.258	1	0.000	0.612	0.612	0.298	1	0.000	0.585	0.585
AD	1.315	1	0.091	0.251	0.363	1.843	1	0.149	0.175	0.233
MD	1.543	1	0.120	0.214	0.363	2.539	1	0.201	0.111	0.222
RD	1.206	1	0.074	0.272	0.363	0.969	1	0.228	0.085	0.222
**Mobility**										
FA	1.892	1	0.124	0.169	0.338	0.004	1	0.000	0.948	0.948
AD	0.101	1	0.000	0.751	0.751	1.271	1	0.068	0.260	0.346
MD	0.300	1	0.000	0.584	0.751	2.000	1	0.131	0.157	0.323
RD	2.312	1	0.150	0.128	0.338	1.962	1	0.129	0.161	0.323
**Vibration sensation**										
FA	1.009	1	0.013	0.315	0.833	1.017	1	0.018	0.313	0.584
AD	0.660	1	0.000	0.417	0.833	0.790	1	0.000	0.374	0.584
MD	0.174	1	0.000	0.677	0.882	0.602	1	0.000	0.438	0.584
RD	0.022	1	0.000	0.882	0.882	0.263	1	0.000	0.608	0.608

DTI = diffusion tensor imaging, FA = fractional anisotropy, AD = axial diffusivity, MD = mean diffusivity, RD = radial diffusivity, DF = degree of freedom

LURN Symptom Index-10 (LURN SI-10)

Instruction: This questionnaire asks you about different urinary symptoms. Please read each question carefully, and then select the response that best describes your symptoms.

	**Never**	**A few times**	**About half the time**	**Most of the time**	**Every time**
1. In the past 7 days, how often did you feel a sudden need to urinate?	0	1	2	3	4
2. In the past 7 days, how often did you leak urine or wet a pad after feeling a sudden need to urinate?	0	1	2	3	4
3. In the past 7 days, how often did you leak urine or wet a pad while laughing, sneezing, or coughing?	0	1	2	3	4
4. In the past 7 days, how often did you leak urine or wet a pad when doing physical activities, such as exercising or lifting a heavy object?	0	1	2	3	4
5. In the past 7 days, how often did you have pain or discomfort in your bladder while it was filling?	0	1	2	3	4
6. In the past 7 days, how often did you have a delay before you started to urinate?	0	1	2	3	4
7. In the past 7 days, how often was your urine flow slow or weak?	0	1	2	3	4
8. In the past 7 days, how often did you dribble urine just after zipping your pants or pulling up your underwear?	0	1	2	3	4
9. In the past 7 days, during waking hours, how many times did you typically urinate?	0 (3 or fewer times a day)	1 (4-7 times a day)	2 (8-10 times a day)	3 (11 or more times a day)	
10. In the past 7 days, during a typical night, how many	0 (none)	1 (1 time)	2	3 (More than 3 times)	

times did you wake up and urinate?			(2-3 times)	

LURN Symptom Index-29 (LURN SI-29)

Instruction: This questionnaire asks you about different urinary symptoms. Please read each question carefully, and then circle the response that best describes your symptoms.

Section A

	**Never**	**A few nights**	**About half the nights**	**Most nights**	**Every night**
6. In the past 7 days, how often did you leak urine during the night, including wetting a pad or the bed?	0	1	2	3	4

Section B

	**Never**	**A few times**	**About half the time**	**Most of the time**	**Every time**
9. In the past 7 days, how often did you have pain or discomfort while urinating?	0	1	2	3	4

Section C

	**Never**	**A few times**	**About half the time**	**Most of the time**	**Every time**
11. In the past 7 days, how often did you have to push when urinating?	0	1	2	3	4
13. In the past 7 days, once you started urinating, how often did your urine flow stop and start again?	0	1	2	3	4

Section F

	Never	A few times	About half the time	Most of the time	Every time
25. In the past 7 days, how often did you feel that your bladder was not completely empty after urination?	0	1	2	3	4
